# Evaluation of the therapeutic effect of *pomegranate peel ginger* ultrafine powder on chronic enteritis in mice by regulating intestinal microbiota

**DOI:** 10.3389/fimmu.2025.1563225

**Published:** 2025-03-17

**Authors:** Zhenhuan Guo, Xiaohua Wang, Yupeng Li, Yanling Zhang, Peng Guo, Jicheng Zhang, Zhiqiang Zhang, Xia Ma

**Affiliations:** ^1^ Zhengzhou Key Laboratory of Immunopharmacology of effective components of Chinese Veterinary Medicine, Henan University of Animal Husbandry and Economy, Zhengzhou, Henan, China; ^2^ College of Animal Science and Technology, Foshan University, Foshan, Guangdong, China; ^3^ Department of pharmacology, Henan University of Traditional Chinese Medicine, Zhengzhou, Henan, China

**Keywords:** ginger, pomegranate peel, ultrafine powder, chronic enteritis, gut microbiota

## Abstract

To explore the efficacy and mechanism of *Pomegranate peel Ginger ultrafine* powder (PG) in treating chronic enteritis in mice. Sixty SPF-grade mice were randomly divided into a blank group, a model group, loperamide hydrochloride group (5 mg/kg), a high-dose PG group (100 mg/kg), a medium-dose group (50 mg/kg), and a low-dose group (25 mg/kg), with 10 mice in each group and an equal number of males and females. A chronic enteritis mouse model was established using a multifactorial method of low temperature + ice water + castor oil. The blank group was given an equal amount of physiological saline intragastrically, while the other groups were intervened with corresponding drugs for 7 consecutive days. After 7 days, samples were collected, and Enzyme-linked immunosorbent assay (ELISA) was used to detect the levels of interleuckin 1β (IL-1β), IL-6, and Tumor necrosis factorα(TNF-α) in mouse serum. HE staining was used to examine the pathological changes in the small intestine. oxidative reagent kits were used to detect the content of total superoxide dismutase(T-SOD) and Malondialdehyde (MDA) in the small intestine. Western blot was used to detect the expression of Aquaporin 8(AQP8) proteins in the small intestine. Real time quantitative reverse transcription polymerase chain reaction (RT-qPCR) was used to detect the expression of AQP3, AQP4, AQP8, and Sodium/hydrogen exchanger 8 (NHE8) genes in the small intestine. metagenomics was used to detect the gut microbiota in mouse feces. Compared with the model group, all doses of PG groups reduced the levels of IL-1β, IL-6, and TNF-α in mouse serum (P<0.05), improved pathological changes in the small intestine, increased the content of T-SOD in the small intestine tissue, reduced the content of MDA, increased the expression of AQP4 and AQP8 mRNA, and decreased the expression of AQP3 and NHE8 mRNA (P<0.05), increased the expression of AQP8 protein. PG could improve the pathological changes of chronic enteritis in mice, enhance antioxidant capacity, and alleviate diarrhea caused by chronic enteritis by downregulating the expression of intestinal epithelial transport proteins and acute-phase proteins, and altering gut microbiota.

## 1 Introduction

Chronic enteritis, induced by bacterial or viral infections, malnutrition, environmental factors, and other causes, is a chronic digestive tract disease that includes inflammatory bowel disease, radiation enteritis, chronic bacterial enteritis, etc. Clinically, it is often manifested by symptoms such as diarrhea, abdominal pain, and indigestion. In traditional Chinese medicine, chronic enteritis is categorized under “diarrhea,” “dysentery,” and “intestinal fetish,” often caused by external pathogenic invasions, improper diet, and spleen-stomach deficiency (1–3). Currently, Western medicine primarily treats chronic enteritis with antibiotics and anti-diarrheal drugs to control diarrhea and inflammation, but the long-term use or misuse of these drugs can easily lead to adverse reactions of varying degrees (4).

PG is prepared by combining *pomegranate peel* and *ginger*, and then processed through ultrafine grinding. The *peel of pomegranate* is the peel of the deciduous shrub, *Punica granatum*, a traditional Chinese medicine with a long history and wide application, is warm in nature and belongs to the large intestine meridian, with astringent and antidiarrheal properties, stopping bleeding, and deworming (5), and studies have shown that *pomegranate peel* is rich in tannins, flavonoids, and alkaloids, which have great potential in anti-inflammatory, antioxidant, antibacterial, and antitumor properties (6). *pomegranate peel* may treat diarrhea through signaling pathways such as PI3K/AKT (7), and also improve intestinal inflammation in mice by inhibiting inflammatory responses and improving oxidative stress (8). *Ginger* is a perennial herb of the ginger family, and dried ginger is dried and processed from ginger, and after processing, the bias and efficacy of both of them are changed to some extent (9). According to the 2020 edition of the Veterinary Pharmacopoeia of the People’s Republic of China, *ginger* is pungent and hot in nature, belonging to the spleen, stomach, kidney, heart, and lung meridians, has the efficacy of dispel the cold, harmonises yin and yang, and clearing phlegm-dampness in the body (10), it can be effective in anti-inflammatory, antioxidant, antibacterial, and antitumor effects, and it has often been clinically used in treating the cold pains of the stomach, vomiting and diarrhea, the cold limbs with weak pulse and the cold-induced cough (11, 12), Some studies have shown that *ginger* can alleviate ulcerative colitis in rats through the TLR4/NF-κB signaling pathway (13). In recent years, Chinese medicine have been favored by the public for their safety and efficacy, and Chinese herbal medicines, after ultramicro pulverization, not only save herbs on the basis of retaining their active ingredients, but also greatly improve the bioavailability of drugs in the organism (14).

In summary, pomegranate peel and ginger both show great potential in treating diarrhea and enteritis, but there are no reports on combining the two to make ultra-fine powder and exploring their therapeutic effects. This experiment is based on this premise, combining *pomegranate peel* with *ginger* to make ultrafine powder and exploring its efficacy on mice with chronic enteritis.

## 2 Materials and methods

### 2.1 Drugs and chemicals

PG formulation: 100g of pomegranate peel,100g of dried ginger. The medicinal materials were all purchased from Yuzhou Kaixuan Pharmaceutical Co., Ltd., and identified by Henan Kangxing Pharmaceutical Co., Ltd. as genuine, in compliance with the “People’s Republic of China Veterinary Pharmacopoeia” standards. They were ultrafine milled and the particle size was qualified as per the standards by Henan Kangxing Pharmaceutical Co., Ltd. Appropriate amounts of the ultrafine powder were taken and mixed with ultrapure water to prepare suspensions of 2 mg/mL, 4 mg/mLand 8 mg/mLrespectively, stored in 4°C refrigerator.

Castor oil (Lot: C805202) was purchased from Shanghai Macklin Biochemical Technology Co., Ltd. Loperamide hydrochloride ((Lot: S26615) was purchased from Shanghai Yuanye Biotechnology Co., Ltd. TNF-α ((Lot: BPE20220), IL-6 ((Lot: BPE20012), and IL-1β ((Lot: BPE20533) ELISA kits were all purchased from Shanghai Langton Biotechnology Co., Ltd. TRIzon Reagent ((Lot: CW0580S) was purchased from Jiangsu Kangwei Century Biotechnology Co., Ltd. 2×HQ SYBR qPCR Mix ((Lot: ZF502), purchased from Beijing Zhuangmeng International Biological Gene Technology Co., Ltd. RAPI Lysis Buffer (Strong) ((Lot: BL504A), SDS-PAGE Protein Loading Buffer (5X) ((Lot: BL502A), 30% Acr-Bis (Lot: BL513B) were all purchased from White Shark Biotechnology Co., Ltd. cDNA Reverse Transcription Kit ((Lot: MF166-plus), M5 Protease Inhibitor Cocktail (Catalog Number: MF183-01) were purchased from Beijing Huagoumei Biotechnology Co., Ltd. 1.5M Tris-HCL Buffer (pH 8.8) ((Lot: T1010), 1M Tris-HCL Buffer (pH=6.8) ((Lot: T1020), Bovine Serum Albumin V ((Lot: A8020), Skim Milk Powder ((Lot: D8340) were all purchased from Beijing Solaibao Technology Co., Ltd. TEMED ((Lot: ST728), 10% SDS ((Lot: ST628) were both purchased from Shanghai Biyuntian Biotechnology Co., Ltd. Ammonium persulfate (Lot: GC101013), purchased from Wuhan Saiwei Biotechnology Co., Ltd. T-SOD Kit ((Lot: WLA110b), MDA Kit ((Lot: WLA048b),were all purchased from Jiangsu Qinke Biological Research Center Co., Ltd.β-actin antibody ((Lot: AF7018), AQP8 antibody ((Lot: DF9224), NHE8 antibody ((Lot: DF4519), were all purchased from Jiangsu Qinke Biological Research Center Co., Ltd.HRP-Goat Anti-Rabbit IgG ((Lot: ZI412-1) was purchased from Beijing Zhuangmeng International Biological Gene Technology Co., Ltd. PageRuler™ Pre-stained Protein Molecular Weight Standard 10-180kDa (Lot: 26616), purchased from Thermo Fisher Scientific. Methanol ((Lot: MS1922-801) was purchased from U.S. Tiandi Co., Ltd. PVDF Membrane ((Lot: ISEQ00010) was purchased from Merck Biosciences. Primer Synthesis, High Sensitivity ECL Chemiluminescent Detection Kit ((Lot: C500044-0100) was purchased from Shanghai Shenggong Biological Engineering (Shanghai) Co., Ltd.

### 2.2 Animals

Sixty 6-8 week-old SPF-grade Kunming white mice, half male and half female Purchased from Huaxing Experimental Animal Farm in Zhengzhou, Henan Province, they are housed in the Henan University of Animal Husbandry and Economy (Breeding License Number: SYXK(YU)2021-0012), where the breeding conditions are controlled with environmental temperature between 20∼25 °C and humidity between 40%∼70%, with free access to food and water for all animals.

### 2.3 Experimental grouping, model establishment and drug administration method

**Figure 1:**
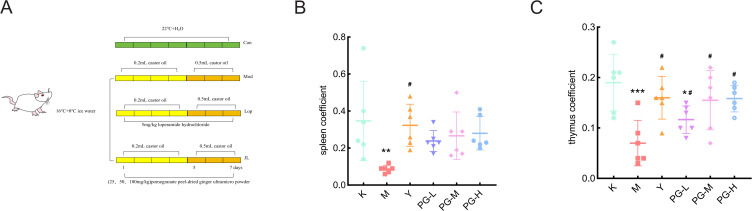
Experimental arrangement and changes in mouse organ index. **(A)** Schematic diagram of the experimental plan. **(B, C)** Mouse organ index. ^★★★^ *P* < 0.001,^★★^ *P* < 0.01,^★^ *P*<0.05, compared with blank control group(K), ^#^ *P*<0.05 compared with model group(M).

After a one-week acclimation period, the mice were randomly divided into six groups, each consisting of 10 mice with an equal number of males and females, namely the blank control group(K), the model group(M), the loperamide hydrochloride group (Y)(5 mg/kg), the high-dose PG group (PG-H)(100 mg/kg), the medium-dose group(PG-M) (50 mg/kg), and the low-dose group (PG-L)(25 mg/kg). Except for the blank control group, which was raised under normal conditions, the rest were housed at 16°C. They were given 0°C ice water to drink freely for 12 hours daily. At 9:00 AM, each mouse was oral gavage with 0.2 mL of castor oil. At 2:00 PM, they were weighed and then oral gavage with the corresponding positive drugs and the appropriate doses of PG suspensions. Referring to the literature and based on the results of the preliminary experiments, from the fifth day of the experiment, the volume of castor oil oral gavage was adjusted to 0.5 mL for three consecutive days (**Figure 1A**) (15, 16).

### 2.4 Measurement indicators

#### 2.4.1 Measurement of daily mental status and body weight in mice

Mouse mental status was observed daily after the start of drug administration, and body mass was measured daily in all mice to observe changes in body weight.

#### 2.4.2 Sample collection and processing

After the last administration of the drug, the mice were fasted but allowed free access to water. The following morning, blood was collected after euthanizing the mice by cervical dislocation, and the blood was allowed to clot and then centrifuged to separate the serum, which was stored at -20°C for later use. The spleen and thymus of the mice were weighed and recorded. A section of the small intestine was fixed in 4% paraformaldehyde, the rest of the small intestine and other tissues were stored in a -80°C freezer. Intestinal contents were collected from the colon and placed into 1.5 mL centrifuge tubes, which were then stored in a -80°C.

#### 2.4.3 The spleen and thymus coefficients

Organ coefficient: organ weight (g)/body weight (g) ×100.

#### 2.4.4 HE staining

Small intestine tissues with obvious pathological changes from each group were fixed with paraffin. The tissues were then cut into continuous sections with a thickness of 4 μm. After drying, the sections were deparaffinized, stained with hematoxylin-eosin, dehydrated again, and then treated with xylene for transparency, followed by mounting. The pathological changes in the small intestine tissues of the mice from each group were observed under a light microscope.

#### 2.4.5 ELISA analysis

Collect mouse serum and strictly follow the ELISA kit instructions for operation. After adding the samples, measure the absorbance at a wavelength of 450nm and determine the concentrations of TNF-α, IL-6, and IL-1β in the serum based on the standard curve.

#### 2.4.6 Detection of SOD and MDA levels in the small intestine tissue

Take mouse small intestine tissue, add an appropriate amount of PBS, homogenize, centrifuge at 4°C at 12,000 rpm for 10 minutes, and take the supernatant to follow the steps in the assay kit manual to detect the levels of SOD and MDA in the intestinal tissue.

#### 2.4.7 Western blot analysis

Cut an appropriate amount of small intestine tissue, grind and homogenize, add pre-prepared RIPA lysis buffer (with protease inhibitors), and let it lysate on ice for 30 minutes, then centrifuge at 12,000 r/min, 4°C for 15 minutes, and take the supernatant. Add SDS-PAGE protein loading buffer and boil to denature, then store at -20°C. Prepare the gel and load samples, keeping the amount consistent per well (10∼30 μg). The voltage for the stacking gel is 80 V for 30 minutes, and for the separating gel is 120 V for 30∼50 minutes. Stop the electrophoresis as soon as the bromophenol blue reaches the bottom of the gel, and perform transfer to a 0.22 μm PVDF membrane at a constant voltage of 85 V for 60 minutes. Immerse the PVDF membrane completely in 5% milk-TBST and incubate with shaking at room temperature for 1 hours. dilute the primary antibodies (β-actin, 1:10000. AQP8) in 5% BSA-TBST and incubate overnight at 4°C. the next day, take out the PVDF membrane, wash with PBST three times, 5 minutes each time. dilute the secondary antibody in 5% milk-TBST (1:7500) and incubate at room temperature for 60 minutes. wash the membrane with TBST three times, 5 minutes each time. after exposure, save and export the image, and analyze the grayscale values using ImageJ professional grayscale analysis software.

#### 2.4.8 RT-qPCR analysis

Total RNA was extracted using the TRIzol method, and after detecting the concentration and purity with a microspectrophotometer, the RNA was reverse transcribed into cDNA. RT-qPCR was performed using the SYBR Green method, with primers designed as shown in **Table 1**.

**Table 1:** Primer sequences for mRNA amplification.

Primer	Sequence (5’->3’)
β-actin	F:TATAAAACCCGGCGGCGCA R:TCATCCATGGCGAACTGGTG
AQP3	F:GGGCCTTTGCCAACAATGAG R:CGGCTGTGCCTATGAACTGA
AQP4	F:CTCTGGGCATCCTGTCACAA R:CAGGAATGTCCACACTTAGACACA
AQP8	F:GGTGAATGTCCCCAGTCCTT R:CTACACATTGGTGTCTCCCCA
NHE8	F:CGTGTGCTTGAAGTCGCATT R:AGGATGATGCAGATGGCTTCTT

#### 2.4.9 metagenomic sequencing

Place the previously collected mouse intestinal contents on dry ice and deliver them to Beijing Research Dog Technology Co., Ltd. for metagenomic sequencing within 24 hours.

### 2.5 Statistical analysis

Each experiment was repeated independently at least thrice. Graphs were drawn using GraphPad Prism 8.0. The differences between relevant groups of data were analyzed using IBM SPSS Statistics 27. One-way ANOVA was used to analyze the differences between three or more sets of data.

## 3 Results

### 3.1 Changes in mental status and body weight of mice

During the experiment, the mice in the blank group were active, had glossy fur, were responsive, and had normal feces. The other groups observed an increase in the frequency of loose stool excretion, with soft, loose, or watery stools after castor oil gavage. There were signs of slow body weight gain, huddling, and lethargy, with a slight decrease in body weight, listless mice, loose fur, and reduced movement, indicating successful model establishment. In the last three days, compared with the blank group, the body weight of the mice in the model group significantly decreased. Compared with the model group, the body weight decrease in the PG groups at various doses and the positive drug group was somewhat slowed (**Table 2**).

**Table 2:** Changes in body weight of mice in each group( $\bar{x}$ ± s, n=6, g).

Groups	Day 1	Day 2	Day 3	Day 4	Day 5	Day 6	Day 7
K	30.66 ± 3.40	31.25 ± 3.70	31.14 ± 3.89	31.18 ± 3.71	31.44 ± 3.08^#^	31.76 ± 2.97^#^	32.01 ± 3.09^#^
M	30.24 ± 2.97	29.93 ± 2.82	29.71 ± 2.80	29.64 ± 2.62	29.26 ± 2.95	29.04 ± 3.00^★^	28.79 ± 2.80^★^
Y	30.30 ± 2.89	30.40 ± 2.59	30.50 ± 2.51	30.12 ± 2.61	29.91 ± 2.45	29.86 ± 2.69	29.73 ± 2.52^★^
PG-L	30.13 ± 2.36	30.65 ± 2.28	30.25 ± 2.17	29.96 ± 2.30	29.52 ± 2.62	29.50 ± 2.45	29.05 ± 2.47^★^
PG-M	31.16 ± 4.78	31.07 ± 4.64	30.88 ± 4.10	30.95 ± 3.48	30.48 ± 3.60	30.31 ± 3.42	30.29 ± 3.58
PG-H	30.98 ± 1.15	31.17 ± 2.07	30.96 ± 1.19	31.23 ± 1.48	31.57 ± 1.49^#^	31.37 ± 1.89^#^	31.38 ± 1.98^#^

^★^ *P*<0.05, compared with blank control group(K), ^#^ *P*<0.05 compared with model group(M).

### 3.2 Changes in organ coefficient in mice

Compared with the blank control group, the spleen coefficient and thymus coefficient of the model group were significantly reduced (*P*<0.05). Compared with the model group, the spleen coefficient and thymus coefficient in the positive drug group and the high, medium, and low dose groups of PG were markedly increased (*P*<0.05), while the difference in the thymus coefficient of the low dose group of PG was not statistically significant (**Figures 1B, C**).

### 3.3 HE staining

**Figure 2:**
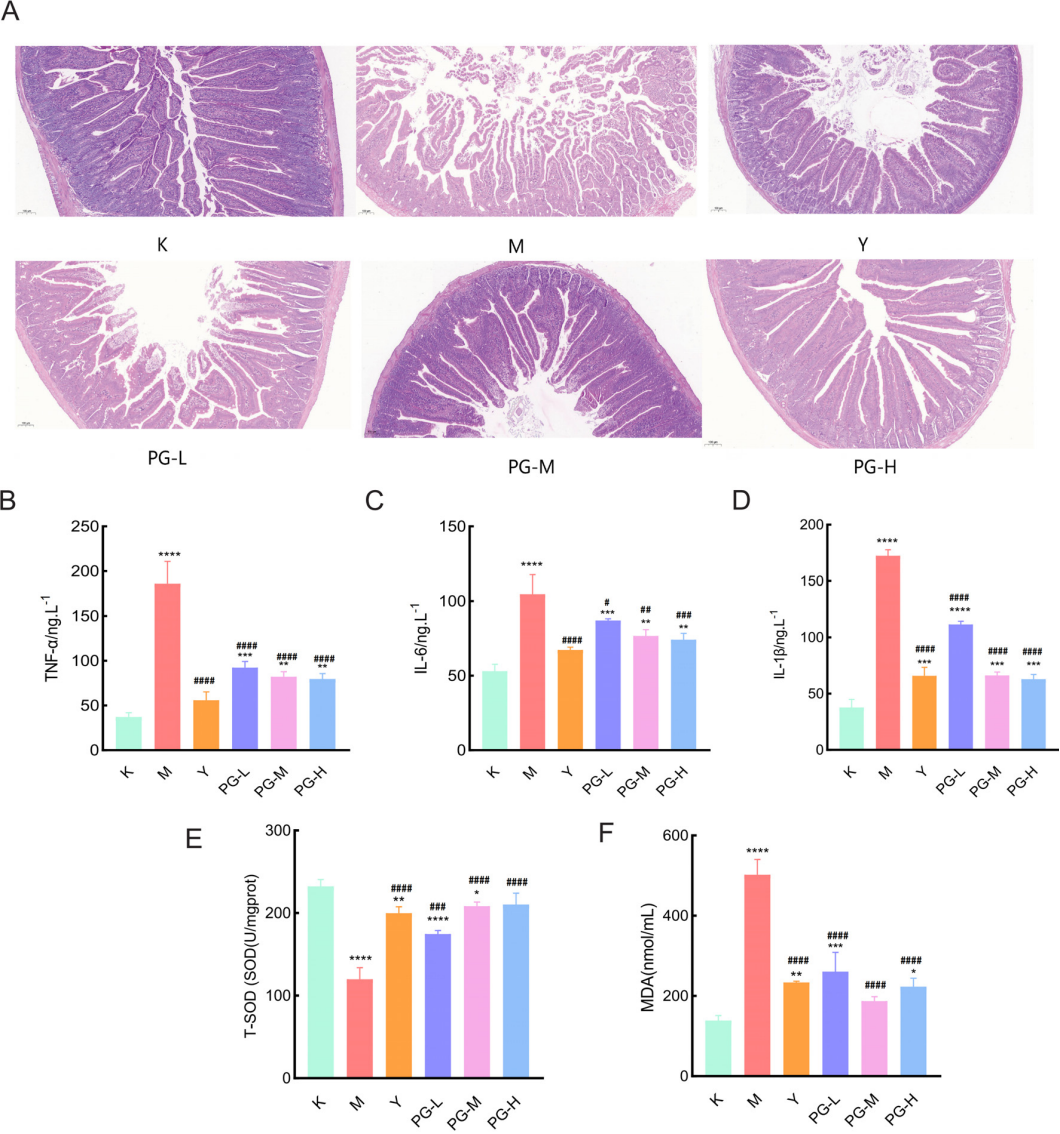
Changes in serum pro-inflammatory factors and antioxidant markers in small intestine tissue. **(A)** Hematoxylin-Eosin (HE) staining of the mouse small intestine (10×10). **(B, C)** The effect of PG on inflammatory factors TNF-α, IL-6, and IL-1β in mice with chronic colitis. **(E)** The effect of PG on T-SOD in mice with chronic colitis. **(F)** The effect of PG on MDA in mice with chronic colitis. ^★★★★^ *P* < 0.0001, ^★★★^ *P* < 0.001,^★★^ *P* < 0.01,^★^ *P*<0.05, compared with blank control group(K). ^####^ *P* < 0.0001, ^###^ *P* < 0.001, ^##^ *P* < 0.01, ^#^ *P*<0.05 compared with model group(M).

The results of hematoxylin and eosin (H&E) staining indicated that the intestinal structure of the blank group was intact and clear, with fewer glands. In the model group, the number of inflammatory cells in the lamina propria of mice increased, the mucosal epithelial cells fell off, the number of glands increased, and a large number of intestinal villi were broken. Compared with the model group, the pathological symptoms of the small intestine in each drug administration group were significantly improved, with less overall damage. Among them, the positive drug group and the medium and high dose groups of pomegranate ginger superfine powder had the best therapeutic effect, with a significant reduction in inflammatory cell infiltration and a marked improvement in the structure of intestinal tissue (**Figure 2A**).

### 3.4 Changes in serum levels of TNF-α, IL-6, and IL-1β

Compared with the blank control group, the levels of TNF-α, IL-6, and IL-1β in the model group were significantly increased (*P*<0.05). Compared with the model group, the levels of TNF-α, IL-6, and IL-1β in the positive drug group and the groups treated with PG were significantly decreased (*P*<0.05) (**Figures 2B–D**).

### 3.5 Changes in T-SOD and MDA levels in small intestinal tissues

Compared with the control group, the level of T-SOD in the tissues of the model group was significantly reduced, and the level of MDA was significantly increased. Compared with the model group, the levels of T-SOD in the positive drug group and the low, medium, and high dose of PG groups were significantly elevated, and the levels of MDA were significantly decreased (**Figures 2E, F**).

### 3.6 Expression of AQP3, AQP4, AQP8, and NHE8 genes in the small intestine

**Figure 3:**
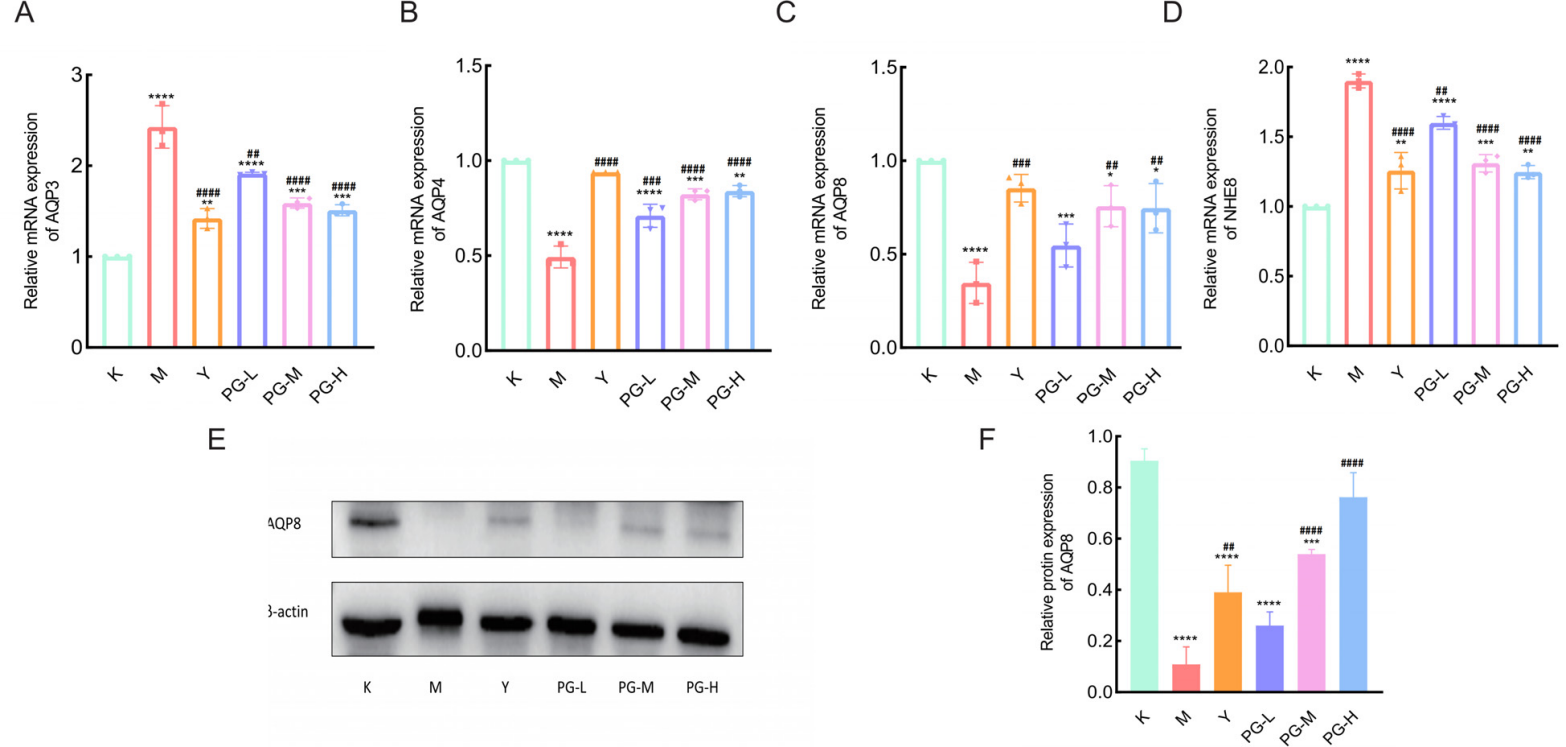
The effect of PG on the expression of water pathway related genes and proteins in the small intestine. **(A-D)** The effect of PG on the gene expression levels of AQP3, AQP4, AQP8, and NHE8 in mice with chronic colitis. **(E, F)** The effect of PG on the protein expression levels of AQP8 in mice with chronic colitis. ^★★★★^ *P* < 0.0001,^★★★^ *P* < 0.001,^★★^ *P* < 0.01,^★^ *P*<0.05 compared with blank control group(K). ^####^ *P* < 0.0001, ^###^ *P* < 0.001, ^##^ *P* < 0.01, ^#^ *P*<0.05 compared with model group(M).

Compared with the control group, the expression levels of AQP3 and NHE8 mRNA in the small intestine of the model group mice were significantly increased (*P*<0.001), while the expression levels of AQP4 and AQP8 mRNA were significantly decreased (*P*<0.001). Compared with the model group, the expression levels of AQP3 and NHE8 mRNA in the loperamide hydrochloride group and the PG groups at various doses were significantly reduced (*P*<0.001), and the expression levels of AQP4 and AQP8 mRNA were significantly increased (*P*<0.001) (**Figures 3A–D**).

### 3.7 AQP8 protein expression in the small intestine

Western blot results showed that compared to the blank group, the expression of AQP8 was decreased in the model group. After interventional treatment with loperamide hydrochloride and various doses of PG, the expression of AQP8 was significantly increased in each treatment group (**Figures 3E, F**).

### 3.8 Macrogenome sequencing

#### 3.8.1 Analysis of species composition

**Figure 4:**
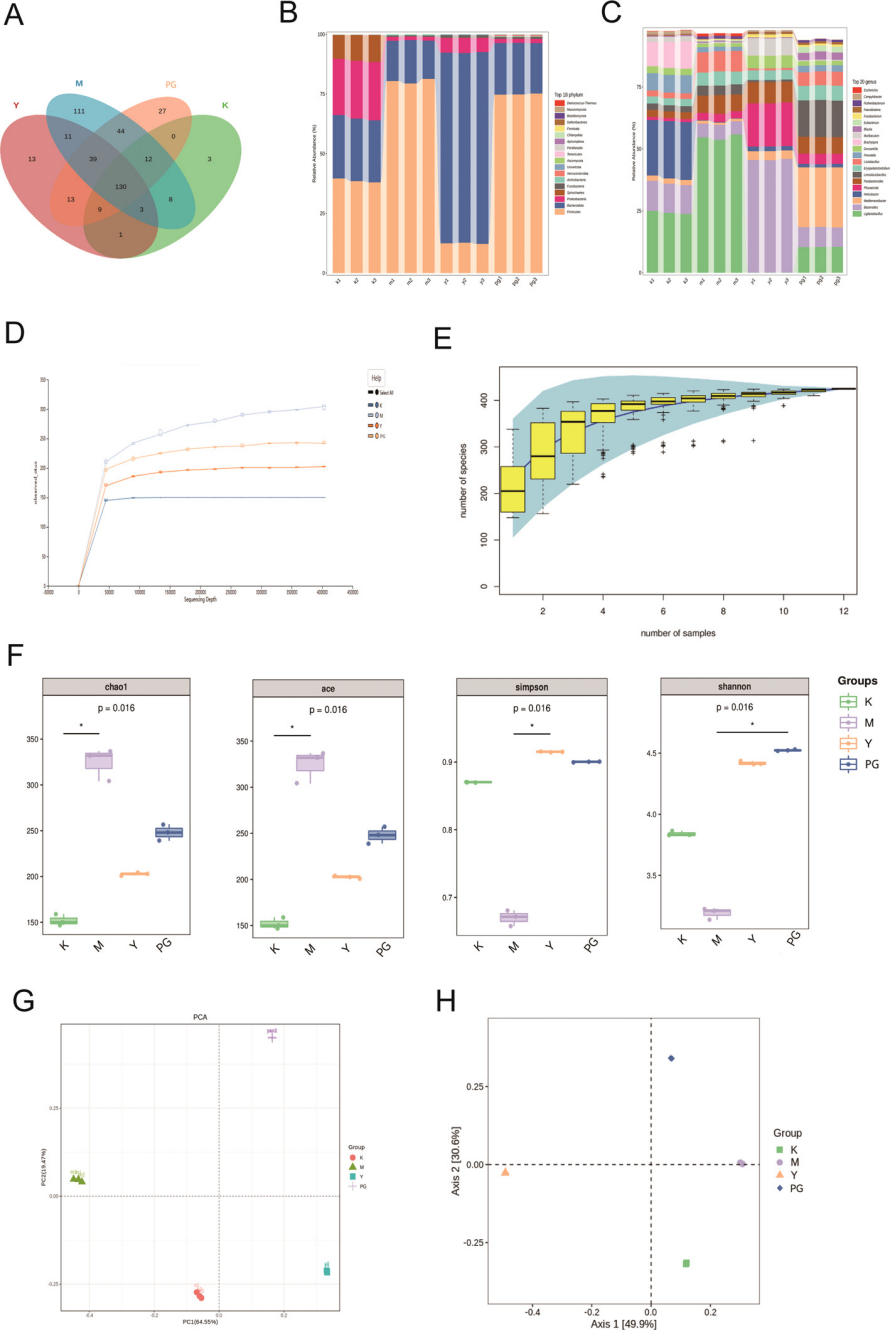
Results of metagenomic sequencing of mice intestinal contents. **(A)** Species Venn analysis. **(B)** Differences in species composition at the phylum level. **(C)** Differences in species composition at the genus level. **(D)** Rarefaction curve. **(E)** Species accumulation curve. **(F)** From left to right, the Chao1 index, ACE index, Simpson index, and Shannon index. **(G)** PCA analysis. **(H)** PCoA analysis. *P<0.05 comparison between groups at both ends of the horizontal line.

To understand the changes in the gut microbiota of mice with chronic enteritis after intervention with PG, we performed high-throughput sequencing on mouse feces. Through Venn analysis of operational taxonomic units (OTUs), we analyzed the similarity and overlap of OTUs composition among different groups. The blank group had 166 OTUs, and the model group had 358 OTUs, which is an increase of 192 OTUs compared to the blank group (**Figure 4A**).

To further explore the differences in species composition at the phylum and genus levels, we analyzed the proportion of sequences at the phylum and genus levels in each sample relative to the total number of sequences (**Figures 4B, C**). At the phylum level, compared to the blank group, the model group showed an increased abundance of *Firmicutes*, while the abundance of *Bacteroidota*, *Proteobacteria*, and *Spirochaetes* decreased. Compared to the model group, the loperamide hydrochloride group had an increased abundance of *Bacteroidota*, and the PG group had an increased abundance of *Firmicutes*. At the genus level, the blank group was predominantly composed of *Ligilactobacillus*, *Bacteroides*, *Helicobacter*, *Brachyspira*, and *Prevotella*. In the model group, *Ligilactobacillus* had the largest proportion, while *Helicobacter*, *Brachyspira*, and *Prevotella* all decreased compared to the model group. *Parabacteroides*, *Ligilactobacillus*, and *Erysipelatoclostridium* increased. In the loperamide hydrochloride group, *Bacteroides* had the largest proportion, followed by *Phocaeicola* and *Parabacteroides*. In the PG group, *Ligilactobacillus* and *Bacteroides* decreased, while *Mediterraneibacter* and *Limosilactobacillus* significantly increased.

#### 3.8.2 Sequencing data quality assessment

The flatness of the sparse curve reflects the impact of sequencing depth on the distribution of observed sample composition. The flatter the curve, the more it indicates that the sequencing results sufficiently reflect the species composition contained in the current sample. Species accumulation curves are also used to measure and predict the extent to which species richness in a community increases with the expansion of sample size, and are widely used to determine whether the sample size is sufficient and to estimate community richness. All group sample curves show a trend of rising first and then flattening, indicating a high richness and evenness of samples, suggesting that the sampling amount is adequate and that experimental analysis can proceed (**Figures 4D, E**).

#### 3.8.3 Alpha diversity analysis

Alpha diversity can indicate the diversity of microbial colonies within a sample. Generally speaking, the larger the Chao1 or ACE index, the higher the microbial richness. Compared with the blank group, the model group showed a significant increase in microbial richness under multifactorial modeling conditions, leading to intestinal microbial disorders in mice, a series of enteritis symptoms, and pathological changes. Compared with the model group, loperamide hydrochloride improved this disorder. The higher the Simpson index value, the greater the microbial diversity. The Shannon index is more sensitive to the richness of the microbial community and rare species, while the Simpson index is more sensitive to evenness and dominant species in the microbial community. Compared with the blank group, the diversity of the model group significantly decreased, while the diversity of the loperamide hydrochloride and PG groups significantly increased, suggesting that loperamide hydrochloride not only regulates the diversity of the intestinal microbiota in chronic enteritis mouse models but also produces rare species, improves inflammation, and regulates the balance of the intestinal microbiota (**Figure 4F**).

#### 3.8.4 Species beta diversity analysis

Beta diversity analysis was conducted to compare the differences in microbial composition among various groups. Based on the compositional structure and abundance information of species in samples at the OTU level, PCA (Principal Component Analysis) and PCoA (Principal Coordinate Analysis) were employed. The microbial composition of each group showed a significant difference compared to the blank group, with the PG treatment group exhibiting the greatest difference from the blank group. This indicates that the intervention with PG enriched the microbial composition of chronic enteritis mice and helped to increase beta diversity (**Figures 4G, H**).

#### 3.8.5 Cluster analysis

**Figure 5:**
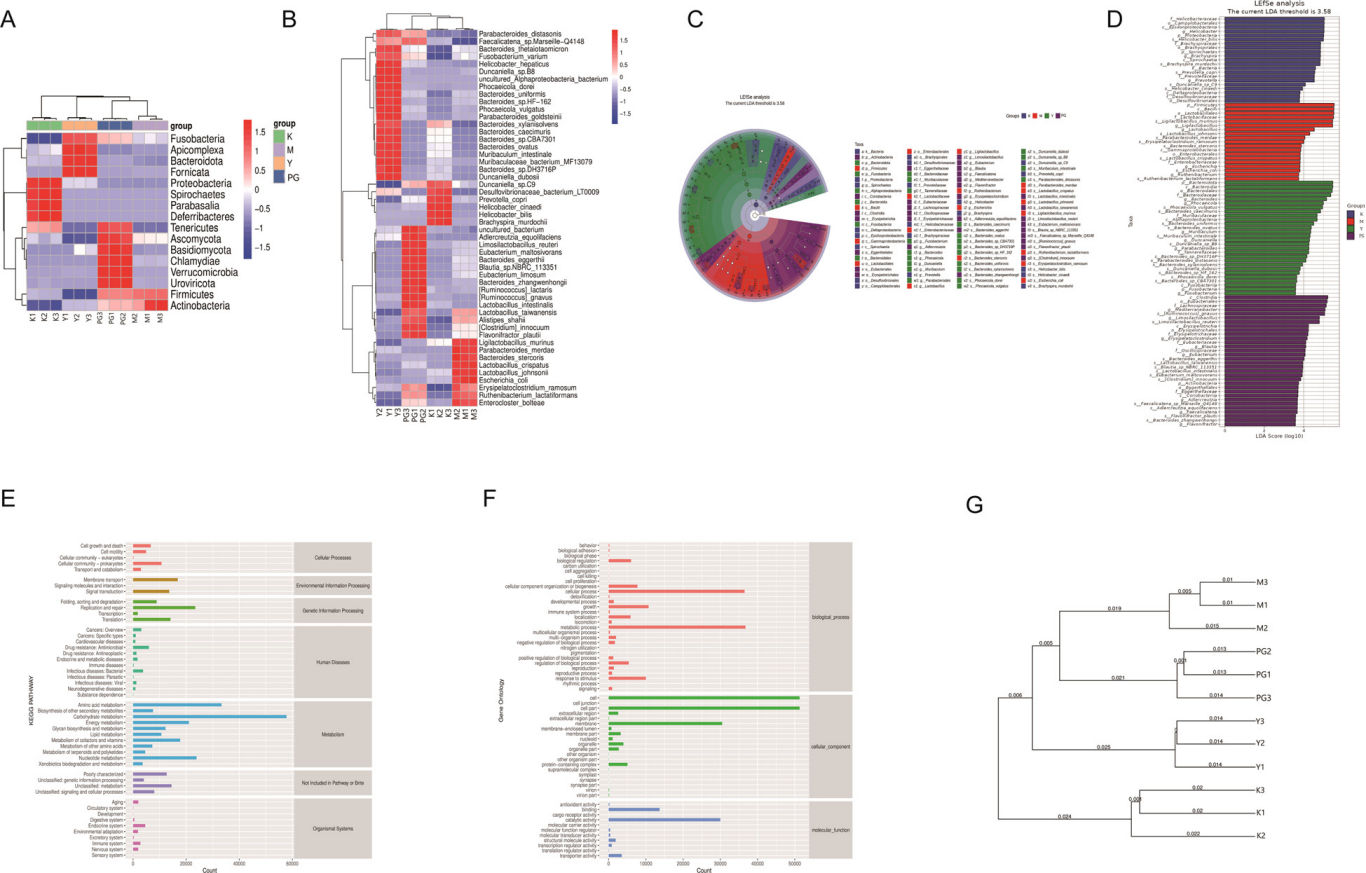
Mice intestinal content clustering analysis and LEfSe analysis results. **(A)** Clustering analysis at the phylum level. **(B)** Clustering analysis at the species level. **(C, D)** LEfSe analysis. **(E, F)** KEGG metabolic pathways. **(G)** UPGMA clustering analysis.

The clustering analysis results indicate that at both the phylum and species levels, the microbial communities of the four groups exhibit unique clustering, with a significant increase in gut microbiota in the PG group, suggesting that PG can significantly enhance the richness of gut microbiota. At the phylum level, the blank group is predominantly found in *Proteobacteria*, *Spirochaetes*, *Parabasalia*, and D*eferribacteres*, the model group in *Actinobacteria* and *Firmicutes*, the metoclopramide hydrochloride group in *Fusobacteria*, *Apicomplexa*, *Bacteroidota*, and *Fornicata*, and the PG group in *Tenericutes*, *Ascomycota*, *Basidiomycota*, *Chlamydiae*, *Verrucomicrobia* and *Uroviricota.* At the species level, the model group is mainly clustered around *Parabacteroides merdae*, *Bacteroides stercoris*, *Lactobacillus crispatus*, *Lactobacillus johnsonii, Escherichia coli, Erysipelatoclostridium ramosum*, *Ruthenibacterium lactatiformans* and *Enterocloster bolteae*. Compared to the model group, the PG group shows distinct clustering in *Faecalicatena* sp. *Marseille*-Q4148, *Bacteroides thetaiotaomicron*, *Fusobacterium varium*, *Helicobacter hepaticus*, *Duncaniella* sp. *B8*, *uncultured Alphaproteobacteria bacterium*, *Phocaeicola dorei, Bacteroides uniformis*, *Bacteroides* sp. HF-162, *Phocaeicola vulgaris*, *Parabacteroides goldsteinii*, *Bacteroides xylanisolvens*, *Bacteroides caecimuris*, *Bacteroides* sp. CBA7301, *Bacteroides ovatus*, *Muribaculum intestinale, Muribaculaceae bacterium* MF13079, B*acteroides* sp. DH3716P and *Duncaniella dubosi* (**Figures 5A, B**).

#### 3.8.6 LEfSe analysis

To identify inter-group differential microbial communities, linear discriminant analysis effect size (LEfSe) was used to recognize differentially abundant taxa (**Figure 5C, D**). The top five significantly differential bacterial genera in the model group are *p_Firmicutes*, *c_Bacilli*, *o_Lactobacillales*, *f_Lactobacillaceae*, and *s_Ligilactobacillus murinus*. For the loperamide hydrochloride group, the top five significantly differential bacterial genera are *p_Bacteroidota*, *c_Bacteroidia*, *o_Bacteroidales*, *f_Bacteroidaceae*, and *g_Bacteroides*. The PG group’s top five significantly differential bacterial genera include *c_Clostridia*, *o_Eubacteriales*, *f_Lachnospiraceae*, *g_Mediterraneibacter*, and s*_Ruminococcus* gnavus.

#### 3.8.7 Functional level KEGG metabolic pathway analysis

The KEGG pathway annotation shows (**Figures 5E, F**) that genes related to metabolism are the most predominant category, especially those involved in amino acids, carbohydrates, and energy metabolism. The Gene Ontology (GO) database is divided into three main categories: Biological Process (BP), Cellular Component (CC), and Molecular Function (MF). The GO pathway results indicate that in terms of BP, the main responses are in cellular process and metabolic process. in terms of CC, the main responses are in cell, cell part, and membrane. and in terms of MF, the main responses are in binding and catalytic activity.

#### 3.8.8 UPGMA cluster analysis

By conducting UPGMA clustering analysis on the four groups of samples, the samples were clustered based on their similarity to each other. The shorter the branch length between samples, the more similar the two samples are. From the figure, it can be seen that the branch distances are closest to the blank group, followed by the loperamide hydrochloride group, the PG group, and the model group. Compared to the blank group, the model group is the farthest, with the PG group and the metoclopramide hydrochloride group being the next closest (**Figure 5G**).

## 4 Discussion

Chronic enteritis is a type of digestive system disease caused by a variety of factors including environment, immunity, and genetics, and the main symptoms are abdominal pain, diarrhea, drainage-like feces (17). In traditional Chinese medicine research, chronic enteritis falls under the categories of “diarrhea,” “dysentery,” and “intestinal fetish,” which are usually caused by factors such as external pathogenic invasions, improper diet, and spleen-stomach deficiency (18). Since enteritis can be induced by multiple factors, the incidence has been gradually increasing in recent years. A substantial amount of research indicates that traditional Chinese medicine can achieve therapeutic effects on chronic enteritis by regulating immunity, signaling pathways, and related gene levels, and it has significant advantages in the treatment of chronic enteritis (19, 20).

Dried ginger is a traditional Chinese medicinal material with a long history, and it has great potential in anti-inflammatory properties. Studies have shown that 6-gingerol and 6-shogaol in dried ginger can improve chemotherapy-induced damage to small intestinal epithelial cells by inhibiting cell pyroptosis mediated by the NF-κB/NLRP3/Caspase-1/GSDMD pathway (21). dried ginger extracts may improve ulcerative colitis by inhibiting the PI3K/Akt/NF-κB signaling pathway (22). *Pomegranate peel* is rich in tannin-like compounds, flavonoids, and other compounds. The tannin-like substances have astringent effects on the intestines, antibacterial and anti-inflammatory properties, and enhance the body’s immune system. Studies indicate that tannin-like substances may improve DSS-induced chronic colitis by inhibiting the expression of JNK/c-Jun genes and the release of pro-inflammatory factors (23).

In this study, mice in the model group continuously exhibited symptoms such as watery diarrhea, depression, coarse fur, and weight loss. Microscopic examination with HE staining revealed inflammatory cell infiltration in the small intestine of the mice, with broken intestinal villi. ELISA results showed significant increases in the levels of inflammatory factors TNF-α, IL-6, and IL-1β in the small intestine of the mice, a significant decrease in T-SOD levels, and a significant increase in MDA levels, indicating successful model establishment. The study indicates that during the occurrence of intestinal inflammation, the levels of oxidative stress markers SOD and glutathione peroxidase (GSH-Px) in the body decrease, while the oxidative product MDA increases (24). The PG ultrafine powder can significantly improve the aforementioned pathological changes in the small intestine of mice and reduce the levels of inflammatory factors TNF-α, IL-6, IL-1β, and MDA in the small intestine of mice.

Diarrhea is usually associated with abnormal water metabolism in the intestine, characterized by an increased water content in feces. Aquaporins (AQPs) are a family of membrane water channels, which are integral, hydrophobic, transmembrane proteins that primarily facilitate the passive transport of water across the membrane by osmotic pressure on both sides of the membrane. Their main function is to regulate the flow of water within and between cells. To date, 13 types of aquaporins have been discovered in humans, widely distributed in specific cell types of various organs and tissues. Based on the transport functions and properties of aquaporins, they are classified into four categories: classic aquaporins, atypical aquaporins, AQP8-type water-ammonia channels, and aquaglyceroporins. In recent years, an increasing number of AQPs subtypes have been found in the digestive system (25). Studies have shown that AQP3 has a broad tissue distribution, and it is permeable to water, glycerol, and urea. It may play a role in cell migration, inflammation, and cancer progression by mediating inflammatory pathways and apoptosis (26, 27). Additionally, AQP3 is mainly expressed in the epithelial cells of the upper digestive tract and plays a crucial role in regulating water transport in the colon (28, 29). AQP4 is present in the basal membrane of crypt and epithelial cells, and AQP4 and AQP8 are highly expressed in colonic tissue, playing an important role in maintaining the integrity of the intestinal mucosa, and both are involved in the absorption and secretion of colonic fluids (30, 31). NaCl is mainly absorbed through the electroneutral pathway mediated by luminal Na+/H+ exchangers (NHEs), and NHE8 is widely expressed in mouse and human skeletal muscle and kidney tissues, as well as in the stomach, duodenum, jejunum, ileum, and colon, making it an important transporter for electroneutral sodium absorption in the intestine (32, 33). Castor oil and its active component ricinoleic acid can reduce the absorption of Na+ and K+ in the small intestine and colon, decrease the activity of Na+, K+ ATPase, alter intestinal permeability, and thus cause diarrhea in mice. The castor oil diarrhea model simulates the physiological changes in mice during colitis-induced diarrhea (34). This study found that PG ultrafine powder can alleviate diarrhea in mice by regulating Na+/H+ exchangers and aquaporins in the small intestine. PG ultrafine powder downregulates the mRNA expression of AQP3 and NHE8 in epithelial cells, upregulates the mRNA expression of AQP4 and AQP8, and alleviates diarrhea in mice by downregulating the mRNA and protein expression of AQP8.

Changes in biological composition and diversity are crucial for promoting inflammation, proliferation, and tumor progression. There is a large and relatively stable microbial community in the animal digestive tract. The diversity of this community can balance the gut microbiota and enhance its adaptability to a variable environment. Both the gut microbes themselves and the short-chain fatty acids they produce play a role in regulating intestinal ion transport and intestinal permeability (35–37). Studies have shown that drugs can alter the gut microbiota to varying degrees during absorption, distribution, metabolism, and excretion. Gut microbes can produce bioactive peptides, including neurotransmitters, the transformation of secondary bile acids, short-chain fatty acids (SCFAs), branched-chain amino acids, and gut hormones, while both acute and chronic stress, anxiety, and depression are influenced by the gut microbiome (38).

Traditional Chinese medicine preparations can significantly improve the composition of the gut microbiota and its dysbiosis, and the gut microbiota may mediate the metabolism of various chemical substances in traditional Chinese medicine (39). The species-level analysis results of this study show that the number of OTUs in both the loperamide hydrochloride group and the ginger pomegranate powder group significantly decreased compared to the model group, indicating that the intervention of ginger pomegranate powder in chronic enteritis mice helps maintain the balance of gut microbial OTUs, while also increasing the diversity of the gut microbiota and restoring a healthy gut environment, as shown in Figure A. Firmicutes and Bacteroidota are the dominant bacterial groups in the gut, accounting for 90% of the gut microbiota. *Bacteroidota* is widely considered to be probiotics, and studies have shown that *Bacteroides* can improve various intestinal diseases, induce the production of the immunomodulatory molecule polysaccharide A by intestinal tissues, and produce anti-inflammatory immune responses, thereby preventing colonic inflammatory diseases (40, 41). The ratio of *Firmicutes* to *Bacteroidota* is widely believed to have a significant impact on maintaining normal intestinal homeostasis. Chronic enteritis reduced the F/B ratio in mice, while PG caused a rebound in the F/B ratio of mice with chronic enteritis induced by ice water and castor oil, indicating that ginger pomegranate powder regulated the dysbiosis of the gut microbiota in mice. In mice with chronic enteritis, the abundance of *Firmicutes* significantly increased, and the abundance of *Bacteroidota* decreased. PG can reverse this change, with the greatest adjustment in α-diversity, leading to better regulation of the gut environment, indicating that PG can significantly alleviate the disorder of the gut microbiota in mice.

In summary, the PG can improve the histopathological changes of chronic enteritis in mice, enhance the antioxidant capacity of mice, and alleviate diarrhea caused by chronic enteritis by downregulating the expression of intestinal epithelial transporters and acute-phase proteins, as well as altering the gut microbiota. However, there are many factors that can induce chronic enteritis in mice, and the occurrence of chronic enteritis is regulated by multiple pathways, so the mechanism of intervention of the PG in chronic enteritis in mice still needs to be further studied.

## 5 Conclusion

From the above we conclude that PG has proved its effectiveness in in the management of chronic enteritis.

## Data Availability

The original contributions presented in the study are included in the article/supplementary material. Further inquiries can be directed to the corresponding author. The original sequence data was submitted to the Sequence Read Archive (SRA) (NCBI, USA) with the accession no. PRJNA1227685.

## References

[1] Qiu Z Song J Zheng XN Jiang X Xu W Ye Q. Research progress in the treatment of chronic enteritis with external treatment of traditional Chinese medicine. Jiangxi J Traditional Chin Med. (2023) 54:73–6. Available online at: https://d.wanfangdata.com.cn/periodical/jxzyy202308024.

[2] Liu Y Hu JJ Pan J Zhang SC Zhang Z. Study on the therapeutic effect and possible mechanisms of Shengyang Chushi decoction on chronic enteritis. World Chin Med. (2021) 16:467–471 + 476. doi: 10.3969/j.issn.1673-7202.2021.03.019

[3] Chen BL Feng YL Wang XF. Wang Xiafang’s experience in treating children’s chronic colitis from the perspective of the spleen. Jiangsu J Traditional Chin Med. (2022) 54:29–32. doi: 10.19844/j.cnki.1672-397X.2022.09.011

[4] Zhang Q Le SJ Chen YY Wang WX Zhao ZB Song YJ. Research progress on traditional Chinese medicine regulating gut microbiota in treatment of chronic diarrhea. Chin Traditional Herbal Drugs. (2022) 53:2539–49. doi: 10.7501/j.issn.0253-2670.2022.08.031

[5] The Pharmacopoeia Committee. Pharmacopoeia of the People’s Republic of China 2020. 2nd ed. China Agriculture Press. (2020) 95.

[6] Zhang J Hu XL Wang H. Research progress in chemical constituent and pharmacological activity of Punica granatum L. J China Pharm Univ. (2023) 54:421–30. doi: 10.11665/j.issn.1000-5048.2023032101

[7] Lu YH Zhang LH Wang JL Zhang X Liu J Liu X. Mechanism of *pomegranate peel* in treatment of diarrhea based on network pharmacology and molecular docking. Drugs Clinic. (2023) 38:2436–43. doi: 10.7501/j.issn.1674-5515.2023.10.008

[8] Lu YX Gao M Li HY Li DF Huang FX Wang L. *Pomegranate peel* extract ameliorates colitis by reducing inflammation and oxidative stress in mice. J Shanxi Med Univ. (2024) 55:50–6. doi: 10.13753/j.issn.1007-6611.2024.01.007

[9] Yang XJ Wang JJ Guo JJ L S Yang ZJ Sui F. Research progress of medicinal properties and efficacy, chemical composition and pharmacological activity of zingiberis rhizoma recens, zingiberis rhizoma and zingiberis rhizoma praeparatum. Traditional Chin Drug Res Clin Pharmacol. (2024) 35:595–605. doi: 10.19378/j.issn.1003-9783.2024.04.018

[10] Committee of Chinese Veterinary Pharmacopoeia. Veterinary Pharmacopoeia of the People’s Republic of China - Part II. China Agriculture Press (2020) 20.

[11] Luo QW Zhong P Meng G Zhang HY. Advance on chemical constituents and pharmacological effects of fresh gingers and dried gingers essential oil. Biol Chem Eng. (2024) 10:184–8. doi: 10.3969/j.issn.2096-0387.2024.03.039

[12] Ye N Wang WS Zhang HL Liu QQ Cai SJ Wu P. The ological effect of dried ginger and its drug progress. Chin Arch Traditional Chin Med. (2024) 42(12):206–9. doi: 10.13193/j.issn.1673-7717.2024.12.042

[13] Yu XC Xie CY Deng SY Xiao HR Ge W. The mechanism of effective chemical components of dried ginger on TLR4/NF-κB signaling pathway in rats with ulcerative colitis based on molecular docking. Chin J Gerontol. (2024) 44:3673–9. doi: 10.3969/j.issn.1005-9202.2024.15.023

[14] Bai XS Song Y Li YQ. Research progress on ultrafine powder of traditional Chinese medicine. Jilin J Chin Med. (2020) 40:1394–6. doi: 10.13463/j.cnki.jlzyy.2020.10.038

[15] Zhu J Yu LQ Fan Y Zhang HN Li FF Li X. Camelina sativa oil treatment alleviates castor oil-induced diarrhea in ICR mice by regulating intestinal flora composition. Evid Based Complement Alternat Med. (2022) 2022:5394514. doi: 10.1155/2022/5394514 35178105 PMC8846971

[16] Meng Y Li WY WuW Wang F Jang XX Yu JY. Research status and review of animal models of cold-dampness syndrome. Modernization Traditional Chin Med Materia Medica-World Sci Technol. (2022) 24:2579–87. doi: 10.11842/wst.20210729009

[17] Guo M Wang X. Pathological mechanism and targeted drugs of ulcerative colitis: A review. Med (Baltimore). (2023) 102:e35020. doi: 10.1097/MD.0000000000035020 PMC10508406 37713856

[18] Wei L Yao LH Zeng JC Xie ZY. Endogenous metabolic changes and disease mechanisms in chronic colitis rats based on metabolomic analysis. J Hubei Univ Med. (2024) 43:252–255 + 337. doi: 10.13819/j.issn.2096-708X.2024.03.005

[19] Lu M Shi XK Cui XH Wang Y. sed on network pharmacology and experimental verification. J Trop Med. (2024) 24:932–938 + 1068. doi: 10.3969/j.issn.1672⁃3619.2024.07.004

[20] Ma YT Du YH Yang DQ. Study on the mechanism of action of mume pill combined with xiaozhiling in the treatment of chronic radiation proctitis based on NF-κB signaling pathway. Henan Traditional Chin Med. (2024) 44:784–9. doi: 10.16367/j.issn.1003-5028.2024.05.0145

[21] Zhang RF Li YQ Zhang GL Chen SQ Bi PP Xian YH. Protective mechanism of 6-gingerol and 6-shogaol against chemotherapy-induced intestinal epithelial cell injury from pyroptosis. Pharmacol Clinics Chin Materia Med. (2021) 37:26–31. doi: 10.13412/j.cnki.zyyl.2021.06.010

[22] Cheng L Gao YY Yao C Li YL Jiang WJ. Improvement effects of Zingiberis rhizoma extract on mice with ulcerative colitis. China Food Additives. (2023) 34:70–7. doi: 10.19804/j.issn1006-2513.2023.08.009

[23] Bai J He L Zhou P Ma GM. Protective effect and mechanism of Tannis in *Pomegranate Peel* on chronic colitis mice model. Chin J Clin Pharmacol. (2021) 37:1341–5. doi: 10.13699/j.cnki.1001-6821.2021.11.010

[24] Deng YS Diao LH Fan YP Li WY Liang TW Huang H. Chinese materia medica by regulating nrf2 signaling pathway in prevention and treatment of ulcerative colitis:A review. Chin J Exp Traditional Med Formulae. 31(01):321–30. doi: 10.13422/j.cnki.syfjx.20250262

[25] Xu L Guo XD Wang WD Li CL. Classification and gene structure of aquaporins. Adv Exp Med Biol. (2023) 1398:1–13. doi: 10.1007/978-981-19-7415-1-1 36717483

[26] Wang Y Guan WX Zhou Y Zhang XY Zhao HJ. Red ginseng polysaccharide promotes ferroptosis in gastric cancer cells by inhibiting PI3K/Akt pathway through down-regulation of AQP3. Cancer Biol Ther. (2024) 25:2284849. doi: 10.1080/15384047.2023.2284849 38051132 PMC10761076

[27] Li H Zhang DD Wang TH Luo XY Xia HY Pan X. Screening the effective components in treating dampness stagnancy due to spleen deficiency syndrome and elucidating the potential mechanism of Poria water extract. Chin J Nat Med. (2023) 21:83–98. doi: 10.1016/S1875-5364(23)60392-9 36871985

[28] Zhan Y Tang X Xu H Tang S. Maren pills improve constipation via regulating AQP3 and NF-kappaB signaling pathway in slow transit constipation *in vitro* and *in vivo* . Evid Based Complement Alternat Med. (2020) 2020:9837384. doi: 10.1155/2020/9837384 32774435 PMC7396072

[29] Ma XW Tang XG. Exploring the mechanism of action of Atractylodes macrocephala on low transit constipation in rats based on AQP3, AQP4, and AQP9. Chin Arch Traditional Chin Med., 1–26.

[30] Cai T Dong Y Feng Z Cai B. Ameliorative effects of the mixed aqueous extract of Aurantii Fructus Immaturus and Magnoliae Officinalis Cortex on loperamide-induced STC mice. Heliyon. (2024) 10:e33705. doi: 10.1016/j.heliyon.2024.e33705 39040398 PMC11261063

[31] Zhao L Xu SS Guo WSLiu P Xi ZW. Effects of probiotics combined with montmorillonite powder on intestinal mucosa and expressions of intestinal microorganism function-related genes in neonatal rats with rotavirus infection. Chin J Clin Pharmacol. (2024) 40:1903–7. doi: 10.13699/j.cnki.1001-6821.2024.13.012

[32] Donowitz M Ming Tse C Fuster D. SLC9/NHE gene family, a plasma membrane and organellar family of Na(+)/H(+) exchangers. Mol Aspects Med. (2013) 34:236–51. doi: 10.1016/j.mam.2012.05.001 PMC3724465 23506868

[33] Sun L Luo C Shen LH Pan ML. A preliminary study on the mechanism of catharsis induced by cinobufagin. Chin J New Drugs. (2023) 32:2311–6. doi: 1003-3734(2023)22-2311-06

[34] Chen WL Peng XY Kang HH Pan YF Tang XG Jiang SJ. Effect and mechanism of Huangqin Decoction in treatment of castor oil-induced diarrhea mice. Chin Traditional Herbal Drugs. (2024) 55:1590–9. doi: 10.7501/j.issn.0253-2670.2024.05.017

[35] Vanjari H Mohan M Saudagar P Guthale G Dalvi M. Protective effect of SKB_Gutbiotic against castor oil and E.coli induced diarrhea in laboratory animals. Microb Pathog. (2020) 143:104078. doi: 10.1016/j.micpath.2020.104078 32142870

[36] Saffouri GB Shields-Cutler RR Chen J Yang Y Lekatz HR Hale VL. Small intestinal microbial dysbiosis underlies symptoms associated with functional gastrointestinal disorders. Nat Commun. (2019) 10:2012. doi: 10.1038/s41467-019-09964-7 31043597 PMC6494866

[37] Vicentini FA Keenan CM Wallace LE Woods C Cavin JB Flockton AR. Intestinal microbiota shapes gut physiology and regulates enteric neurons and glia. Microbiome. (2021) 9:210. doi: 10.1186/s40168-021-01165-z 34702353 PMC8549243

[38] Long-Smith C O'Riordan KJ Clarke G Stanton C Dinan DG Cryan JH. Microbiota-gut-brain axis: new therapeutic opportunities. Annu Rev Pharmacol Toxicol. (2020) 60:477–502. doi: 10.1146/annurev-pharmtox-010919-023628 31506009

[39] Xing C Liu Y Wang SH Zhang J Liu Li N. Regulation of intestinal flora in patients with chronic atrophic gastritis by modified Chai Shao Liu Jun Zi decoction based on 16S rRNA sequencing. Med (Baltimore). (2024) 103:e37053. doi: 10.1097/MD.0000000000037053 PMC10860994 38335441

[40] Cao B Wang SY Li RS Wang ZH Li TF Zhang YY. Xihuang Pill enhances anticancer effect of anlotinib by regulating gut microbiota composition and tumor angiogenesis pathway. BioMed Pharmacother. (2022) 151:113081. doi: 10.1016/j.biopha.2022.113081 35605293

[41] Qi M Chu SWang W Fu X Jiang C Zhang L. Safflower polysaccharide ameliorates acute ulcerative colitis by regulating STAT3/NF-kappaB signaling pathways and repairing intestinal barrier function. BioMed Pharmacother. (2024) 174:116553. doi: 10.1016/j.biopha.2024.116553 38593703

